# Rapid and Non-Destructive Analysis of Corky Off-Flavors in Natural Cork Stoppers by a Wireless and Portable Electronic Nose

**DOI:** 10.3390/s22134687

**Published:** 2022-06-21

**Authors:** José Pedro Santos, Isabel Sayago, José Luis Sanjurjo, María Soledad Perez-Coello, María Consuelo Díaz-Maroto

**Affiliations:** 1 Instituto de Tecnologías Físicas y de la Información (ITEFI), Consejo Superior de Investigaciones Científicas, Serrano 144, 28006 Madrid, Spain; i.sayago@csic.es (I.S.); jl.s.m.3@csic.es (J.L.S.); 2 Food Technology, Facultad de Ciencias y Tecnologías Químicas, Instituto Regional de Investigación Científica Aplicada (IRICA), Universidad de Castilla-La Mancha, 13071 Ciudad Real, Spain; soledad.perez@uclm.es (M.S.P.-C.); mariaconsuelo.diaz@uclm.es (M.C.D.-M.)

**Keywords:** natural cork stoppers, corky off-flavors, electronic nose, machine learning algorithms, artificial neural networks

## Abstract

This article discusses the use of a handheld electronic nose to obtain information on the presence of some aromatic defects in natural cork stoppers, such as haloanisoles, alkylmethoxypyrazines, and ketones. Typical concentrations of these compounds (from 5 to 120 ng in the cork samples) have been measured. Two electronic nose prototypes have been developed as an instrumentation system comprise of eight commercial gas sensors to perform two sets of experiments. In the first experiment, a quantitative approach was used whist in the second experiment a qualitative one was used. Machine learning algorithms such as k-nearest neighbors and artificial neural networks have been used in order to test the performance of the system to detect cork defects. The use of this system tries to improve the current aromatic defect detection process in the cork stopper industry, which is done by gas chromatography or human test panels. We found this electronic nose to have near 100 % accuracy in the detection of these defects.

## 1. Introduction

Cork stoppers are the main closures chosen to seal the wine bottles, and will influence the quality of the wine, mainly those that require long stays in the bottle. For this reason, the cork industry has strict quality systems, the goal of which is the total absence of defects. In this sense, one of the main problems of the cork industry is the detection of the defect known as “cork taint”.

Halogenated aromatic compounds have been identified as the typical cause of the “cork taint” defect, specifically the 2,4,6-tricloroanisol (TCA) and, to a lesser extent, 2,4,6-tribromoanisol (TBA) and 2,3,4,6-tetracloroanisol (TeCA) [1,2]. However, apart from these halogenated derivatives, cork presents other volatile compounds with negative effects such as geosmin, with a strong smell of mold and wet earth, guaiacol with phenolic olfactory notes, 1-octen-3-ol and 1-octen-3-one, with a strong mushroom and earthy odor, or 2-methoxy-3,5-dimethylpyrazine (MDMP) with a characteristic musty and moldy odor [3,4,5].

Most of the cork industries have a gas chromatography system coupled with mass spectrometry, which allows the quantification of different corky off-flavors in cork stoppers. This technique needs a previous step of sample preparation, usually destructive and time-consuming, that sometimes requires the use of organic solvents, such as purge and trap, solid phase microextraction, soxhlet extraction, stir bar sorption, or pressurized fluids [5,6,7].

The control of the presence of typical and atypical compounds responsible for “cork taint” by chromatographic techniques is very useful, although due to their time-consuming and destructive character, only can be applied to a representative sample of cork stoppers and cannot be included in the production lines. Also, some industries have a system for detecting olfactory defects by the human sense, with a great degree of subjectivity. In this sense, it is of special interest to introduce in the quality control of the cork industry a non-destructive analytical technique of the aroma that allows the rapid and precise analysis of many cork samples in a relatively short time. Among the available techniques, the electronic nose (Enose) stands out. An Enose is an instrument composed of a set of chemical gas sensors with partial specificity and an appropriate pattern recognition system, capable of recognizing simple or complex odors [8]. These sensors can be used for the analysis of volatile compounds in different matrices, by transducing chemical signals into electrical signals [9]. In the bibliography, there are several studies about the use of this analytical tool in the wine industry, including aging control, wine discrimination, or grape ripening monitoring [10,11,12]. However, there are practically no studies on their use in the cork industry. It is worth highlighting the determination of TCA in wines using a coupled system of headspace and mass spectrometry [13], or the use of a miniaturized sensor module to detect TCA in cork stoppers [14].

In this work, the feasibility of a small wireless and portable Enose (WiNOSE 6), capable of measuring up to eight microsensors, to detect typical and atypical off-flavor compounds in natural cork stoppers, has been studied. The selected compounds belong to three different chemical families, haloanisoles, alkylmethoxypyrazines and ketones, TCA, MDMP, and 1-octen-3-one, and they present a strong musty, moldy and earthy odor. The concentrations chosen to contaminate the corks were selected according to bibliographic data. Problematic batches of corks had until 14.0 ng per cork of TCA, while batches considered acceptable had values between 2.8 and 4.7 ng per cork [15]. Regarding MDMP, a value of less than 5 ng per cork is considered to present no risk of contamination, while concentrations greater than 15.1 ng per cork are a significant risk to the wine [5]. Finally, for 1-octen-3-one, similar concentrations were selected. This compound was chosen since it is one of the most frequent off-odor, after TCA, detected in tainted wines [16].

## 2. Materials and Methods

### 2.1. Cork Samples

Flower quality natural cork stoppers without sensorial deviant odors were kindly provided by Gruart La Mancha S.A. (Valdepeñas, Ciudad Real, Spain). The cork stoppers became contaminated, by triplicate, with increasing amounts (5, 15, 30, and 60 ng per cork) of 2,4,6-thichoroanisole (TCA) (Merck KGaA, Darmstadt, Germany), 2-methoxy-3,5-dimethylpyrazine (MDMP) (Enamine Ltd., Kyiv, Ukraine), and 1-octen-3-one (Merck KGaA, Darmstadt, Germany). In the case of 1-octen-3-one, the cork stoppers became contaminated with 5, 15, 30, 60, and 120 ng per cork. To reduce the atmospheric oxygen and prevent the volatile components from evaporation, once the cork stoppers were contaminated, they were stored under a vacuum in 150 µm thick food vacuum bags (TED packaging, Dongguan, China) until their analysis.

### 2.2. Electronic Nose

**Figure 1:**
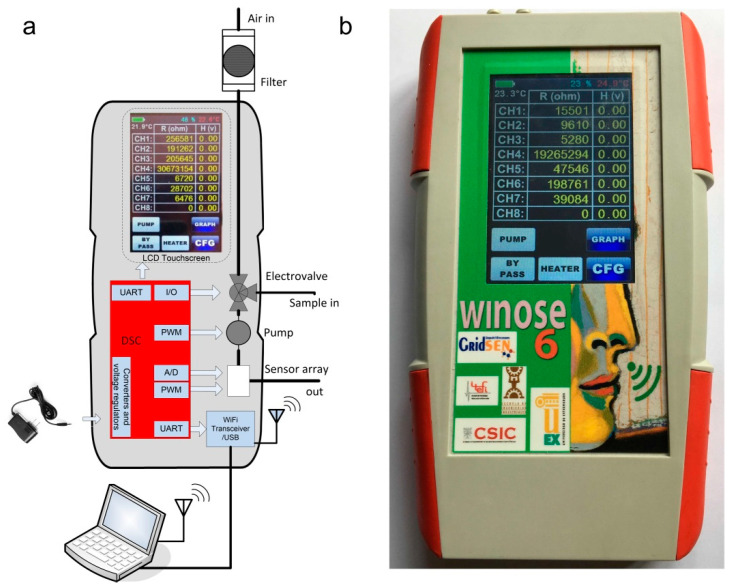
Wi NOSE 6: (**a**) Schematic; (**b**) photograph.

A schematic and a photograph of the Enose used (WiNOSE 6) are shown in Figure 1.

The Enose is a handheld instrument that has the possibility to measure up to eight resistive microsensors. It is a stand-alone instrument with all the fluidics elements to take samples from the outside (pump, electrovalve). A disposable filter allows a clean air path to establish a baseline for the sensors. Temperature and humidity are measured both inside the sensors chamber and outside the Enose. Depending on the application, several sets of sensors can be used. For this study, we have used microsensors from several manufacturers: MICS (SGX Sensortech, Bern, Switzerland), CCS (AMS, Premstaetten, Austria), and TGS (Figaro Engineering Inc, Osaka, Japan). We have used two prototypes each one with its own sensor configuration (Table 1). The sensors have integrated heaters, capable of reaching 500 °C with low power consumption (typically from 10 to 80 mW).

The instrument can be operated through its LCD touch screen but in this work, we controlled them by a PC with a home developed LabVIEW program (version 18, National Instruments, Austin, TX, USA). Measurements are stored both internally on a uSD card and externally on the PC.

### 2.3. Measurement Protocol

We used three replicates of the corks for each defect concentration. Corks were introduced in 50 mL vials with two orifices in the top, one for atmospheric air and the other connected to the Enose. Each measurement cycle consists of a desorption phase of 9 min followed by an adsorption phase of 1 min. For each sample, cycles are repeated several times. Sample measurements were randomized during the experiments. We used a randomized block design (RBD): the samples are divided into three groups or blocks, one for each replicate. Separate randomization is used in each block generating a table of random numbers. In our case, each block is composed of 14 cork samples (1 blank and 13 contaminated corks). We used two Enose prototypes to perform two different experiments. In the first experiment, a quantitative approach was used. We measured all concentrations of cork contaminants. The scope was to use the Enose in such a way as a traditional analytical instrument with numerical output values. In the second experiment, a qualitative approach was used. We only measured the maximum concentrations of contaminants. In this case, the scope was to have a quick field instrument to assess if the cork has a defect and the type of defect. The offline data processing means that the data are saved for further analysis on another PC with the appropriate software while online data processing means that the data are processed inside the Labview program that controls the Enose. Once trained the program will automatically classify each measurement. In the offline experiment, 8 measurements were performed for each class while in the online experiment 15 measurements were obtained for each class.

The sensor responses were calculated as the relation between the resistance value at the end of the air cycle, *Ra*, and the equilibrium resistance value at the end of the sample cycle *Rs*:

*r* = *Ra*/*Rs* (1)

### 2.4. Data Processing

Several multivariate data processing techniques have been used: a linear unsupervised one, principal component analysis (PCA) and nonlinear supervised K-nearest neighbors (kNN) [17], and two types of artificial neural networks: multilayer feed forward neural network (MLFF) [18] and Radial basis neural networks (RBF) [19].

A principal component analysis is a technique included in the set of unsupervised learning algorithms used to describe a multivariate data set by converting the original data set that may be related to each other into new uncorrelated variables. This technique is used to reduce dimensionally the feature matrix. In addition, it allows us to visualize the structure of the data using graphs, which allows us to have a better understanding of the data set or some of the variables separately. Therefore, with this algorithm, we obtain a vector of data of smaller dimensions than the original one and more descriptive variables, called principal components. The principal components are chosen in such a way that in the smallest number of these reside the highest possible variance, being orthogonal to each other, which means that there is no correlation between each component. Usually, a few principal components are used to train the artificial neural networks in order to reduce the computational time. In our case, the three first principal components are used.

To evaluate the performance of the multivariate techniques they have to be validated. Usually, the data set is divided into three sets: training set, to adjust the algorithm parameters during the training stage; cross-validation set, to adjust the algorithm parameters during the training stage and evaluation set, to evaluate the final model. When there are few data available k-fold cross-validation is used. In its simplest form, cross-validation consists of dividing the data set into k sets. Of these, one plays the role of the evaluation set and the rest are merged to play the role of the training set, resulting in a model that is evaluated with a metric. This process is repeated k times, so that each set plays the role of an evaluation set, and k models are produced, each with a different score. To evaluate the effectiveness of the system, the scores of the k models obtained are weighted. In the case that k equals the total number of measurements we call it leave one out cross-validation [17].

Some algorithms reduce the classification and clustering problems to optimization and minima search problems. To facilitate convergence and the correct fitting of the model parameters, a prior operation is to rescale the sample variables in what is known as data normalization. Depending on the range, algorithm, and structure of the data, one normalization or another is used. It is common to work with data that give mean µ = 0 and standard deviation σ = 1 to avoid very uneven data. In this case, the normalized response *r_i_* is calculated as:

(2) $$r_{i}=(r-\bar{r_{i}})/\sigma_{i}$$

where the subscript *i* refers to the *i*-th variable.

In the offline experiment, PCA was performed by OriginPro (2019 version, OriginLab Corporation, Northampton, MA, USA), and kNN, RBF, and MLFF were performed in Matlab (version 12, Mathworks Inc., Natick, MA, USA).

In the online experiment, all the classification algorithms were written in Labview using the Machine Learning Toolkit (MLT) [20].

## 3. Results

**Figure 2:**
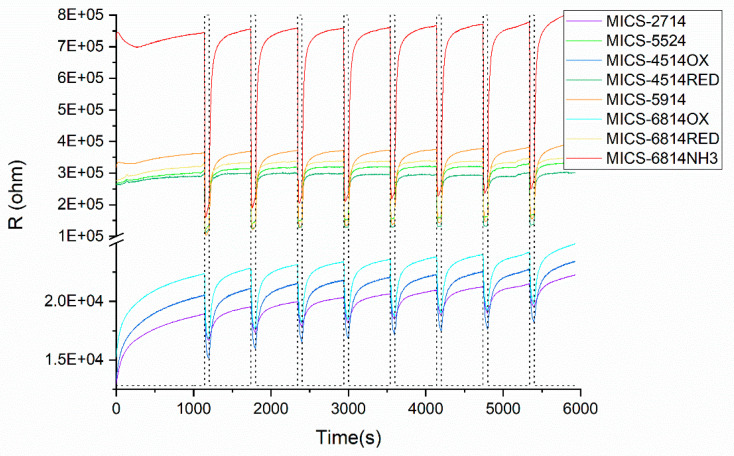
Resistance variations of sensors exposed to blank cork samples. Dashed lines correspond to the sampling electrovalve status (ON/OFF).

In order to test the stability of the sensors, we show, as an example, in Figure 2 resistance variations of set 1 sensors exposed to blank cork samples during 8 measurement cycles.

Resistances are quite stable during the eight measurement cycles although some drift can be observed for the low resistance sensors (MICS-2714, MICS-4514OX, and MICS-6814OX).

PCA analysis showed that more than 90% of the variance is explained by the first two principal components arriving at 99% taking into account the first three principal components in all experiments. The curves with a 90% probability are represented as ellipses in the score plots assuming a Gaussian data distribution.

**Figure 3:**
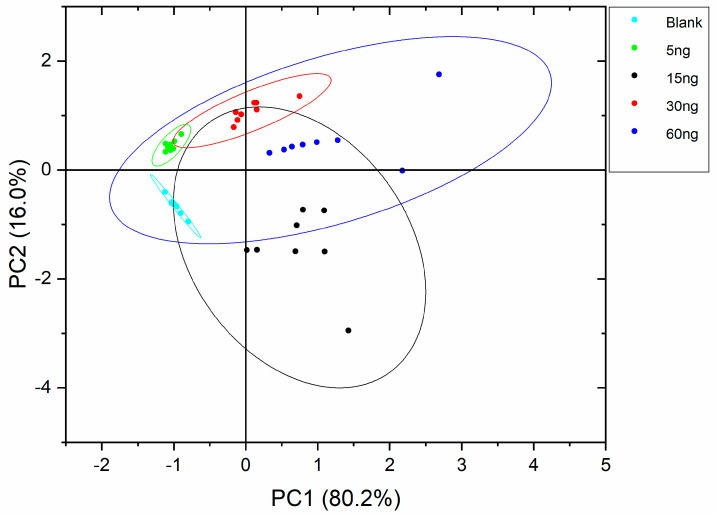
PCA score plot for MDMP.

**Figure 4:**
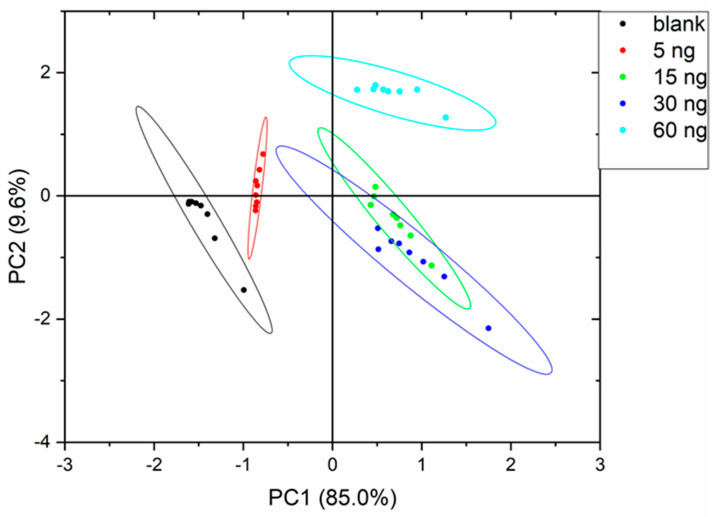
PCA score plot for TCA.

**Figure 5:**
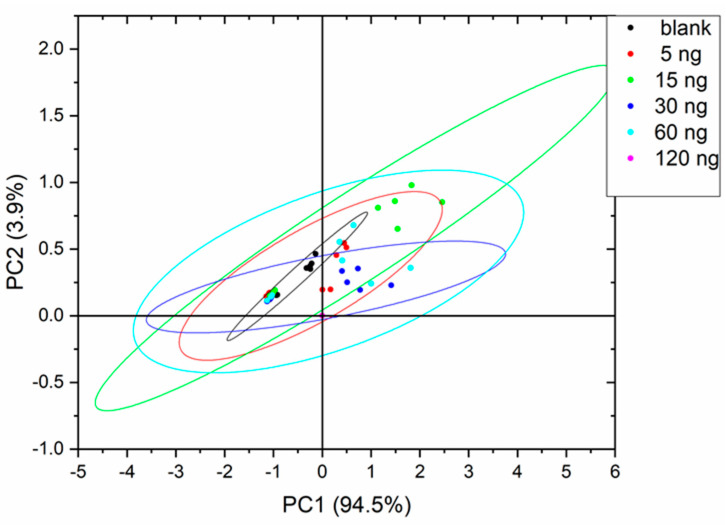
PCA score plot for 1-octen-3-one.

Figure 3, Figure 4 and Figure 5 show the PCA score plots of the two principal components for the three defects.

In general, data are more dispersed for the intermediate defect concentrations and, in fact, this will be reflected in the machine learning classification results. From the above plots, a better separation of classes was obtained for MDMP and TCA. The poor class separation among classes for 1-octen-3-one will lead to the worst performance of the classifiers.

**Figure 6:**
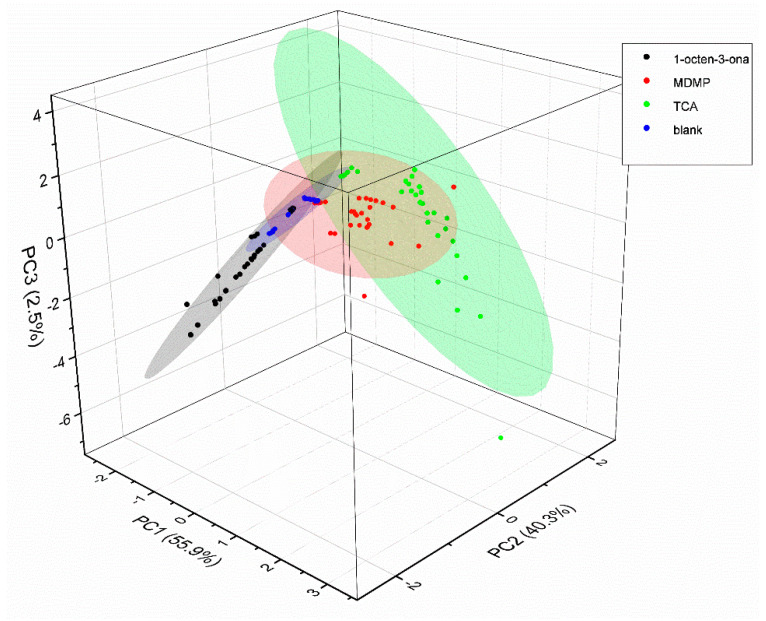
PCA score plot for all measurements.

Figure 6 shows the PCA score plot for all the measurements considering each defect as a separate class. In this case, a 3D representation has been chosen for clarity.

In order to evaluate the performance of the different models, we have to calculate the success rate i.e., the percentage of corrected classified samples. This rate can be obtained from the confusion matrix. In the confusion matrix, the actual values are in the rows whilst the predicted value are in the columns. Therefore, corrected predicted values are in the diagonal of the matrix. The success rate is calculated by dividing the number of correct classified values by the total number of values. This term is often called accuracy. If each class corresponds to a numerical value (defect concentration), we calculated the quantitative success rate. For example, Table 2 shows the confusion matrix of the TCA measurements processed with the RBF neural network. From the diagonal, the correct classified measurements are 39 and the total number of measurements is 40, so the success rate is 97.5%.

If we consider each defect a separated class independently of their concentration we calculate a qualitative success rate.

### 3.1. Offline Experiments

The success rate are shown on Table 3.

We can see we obtained very good accuracy (near 100%) for the quantitative case for the MLFF algorithm except in the case of the 1-octen-3-one, which obtained a 77% success rate. For the qualitative case, both MLFF and RBF algorithms reached near 100% accuracy for all compounds. kNN performed well for the qualitative case but it was not accurate for the quantitative one except for the detection of MDMP.

### 3.2. Online Experiments

In this experiment, the Enose uses the set 2 sensor configuration. Only the maximum concentration of each compound is measured. In this case, we have enough measurements to split the data in train + validation (70%) and test (30%). The multivariate data processing techniques have been developed in Labview in order to integrate the measurement, control, and data processing in one program. The qualitative success rate is 100% for all techniques. The quantitative success rate is shown in Table 4.

As can be seen in Table 4, MLFF is the best classification technique, as in the offline experiment, although the other two give also an excellent performance. Once selected the best technique for the application the system is ready to perform measurement classification in real time. In order to test the performance of the system, we made a simplified triangle test which is a discriminative method used in sensory science to assess if an overall difference is present between two products [21]. In this test, we used the blank sample (B) and the TCA sample (T) The Enose measured the samples in the following order: BBT, BTB, and TBB. The system gave the correct answer for all the sequences.

Very low concentration and high volatility of typical and atypical corky off-flavors, together with the matrix effect, make the determination of these compounds in corks a really difficult task. Gas chromatography-mass spectrometry (GC-MS) has been the most used analytical technique [7]. However, this technique does not allow on-site monitoring since it requires a previous step to extract volatile compounds, which is normally tedious and time-consuming, and sometimes requires high volumes of organic solvents [4,5,7]. There are several direct methods for the analysis of corky compounds in corks by GC, which are normally solvent-free, although some require heating and usually grinding of the corks, therefore only a small fraction of the stopper is analyzed [22,23,24]. For these reasons, different several cork industries have included in their quality control system sensorial methods as an alternative to GC for fast screening of corks. Sensory analysis requires a hard prior training of the tasters to reduce the subjectivity of the results [25]. In addition, it does not allow the analysis of a large number of samples since it implies significant fatigue of the tasters.

The Enose used has significant advantages over traditional analytical and sensorial methods. It is a rapid and efficient alternative for on-site monitoring, requires no heating or sample preparation, and allows whole corks to be analyzed. The results demonstrate the ability of this Enose to differentiate natural corks contaminated with different concentrations of TCA or MDMP, even as low as 5.0 ng. This is especially interesting since both compounds are the main ones responsible for the olfactory defect in wines called “cork taint”. MDMP has a low odor threshold (2.1 ng/L in white wine) and a great affinity for wine, even higher than TCA, with a detection threshold of 4.3 ng/L in white wine [4,5,25]. In the case of MDMP, it is considered a very low risk of wine contamination if its concentration in the cork is less than 5.0 ng [5]. While for TCA, a mean percentage of migration from cork to wine of 4.7% [26] has been estimated, which shows that the Enose can detect TCA in corks at concentrations below those that could cause olfactory defects in wines.

## 4. Conclusions

The nose used in this work, composed of non-specific cross-sensitivity sensors that respond to a wide variety of compounds, can be applied in the wine industry to provide qualitative information about the sample and predict or detect the presence of cork-associated anomalies. The electronic nose has many advantages over traditional methods (gas chromatography, test panels), mainly its speed and the absence of sample preparation. In addition, this sensory method could easily be applied to other beverages where cork odors or other off-flavors might be present. The system presented here has been demonstrated to be very useful in the detection of aromatic defects in natural cork stoppers. We obtained near 100% classification rates in the discrimination of defects such as MDMP, TCA, and 1-octen-3-one. Real time sample classification has been developed with this prototype.

Electronic noses will probably not completely replace complex analytical instruments, but it offers fast real time detection and discrimination solutions and opens the way for their adaptation and integration into the internet of things.

## Figures and Tables

**Table 1:** Sensor setup.

Set 1	Set 2
MICS–2714	CCS801
MICS–5524	CCS803
MICS–4514–OX	MICS–4514–OX
MICS–4514–RED	MICS–4514–RED
MICS–5914	TGS8100
MICS–6814–OX	MICS–6814–OX
MICS–6814–RED	MICS–6814–RED
MICS–6814–NH3	MICS–6814–NH3

**Table 2:** Confusion matrix for the TCA measurements with the RBF neural network. Actual values (rows); predicted values (columns).

	Blank	5 ng	15 ng	30 ng	60 ng
**Blank**	8	0	0	0	0
**5 ng**	0	8	0	0	0
**15 ng**	0	0	7	1	0
**30 ng**	0	0	0	8	0
**60 ng**	0	0	0	0	8

**Table 3:** Success rate for the different pattern recognition techniques for the offline experiments. Units in percentage.

Defect	Qualitative			Quantitative		
	kNN	MLFF	RBF	kNN	MLFF	RBF
**MDMP**	100	100	100	98	100	100
**TCA**	98	100	100	82	100	98
**1-octen-3-one**	90	97	97	49	77	54
**All**	85	99	99	50	98	95

**Table 4:** Success rate for the different pattern recognition techniques for the online experiments. Units in percentage.

Method	Success Rate
**kNN**	96
**MLFF**	99
**RBF**	98

## Data Availability

Not applicable.
